# A stochastic mathematical model for coupling the cytosolic and sarcoplasmic calcium movements in diseased cardiac myocytes

**DOI:** 10.1007/s00249-022-01617-w

**Published:** 2022-09-18

**Authors:** Serife Arif

**Affiliations:** Academic Section, University of Essex, Wivenhoe Park, Colchester, CO4 3SQ UK

**Keywords:** Cardiac myocyte, Ca^2+^ sparks and blinks, Ca^2+^-induced Ca^2+^ release, Stochastic Ca^2+^ release, Stochastic partial differential equations

## Abstract

Several computational studies have been undertaken to explore the Ca^2+^-induced Ca^2+^ release (CICR) events in cardiac myocytes and along with experimental studies it has given us invaluable insight into the mechanism of CICR from spark/blink initiation to termination and regulation, and their interplay under normal and pathological conditions. The computational modelling of this mechanism has mainly been investigated using coupled differential equations (DEs). However, there is a lack of computational investigation into (1) how the different formulation of coupled DEs capture the Ca^2+^ movement in the cytosol and sarcoplasmic reticulum (SR), (2) the buffer and dye inclusion in both compartments, and (3) the effect of buffer and dye properties on the calcium behaviour. This work is set out to explore (1) the effect of different coupled formulation of DEs on spark/blink occurrence, (2) the inclusion of improved sarcoplasmic buffering properties, and (3) the effects of cytosolic and sarcoplasmic dye and buffer properties on Ca^2+^ movement. The simulation results show large discrepancies between different formulations of the governing equations. Additionally, extension of the model to include sarcoplasmic buffering properties show normalised fluorescent dye profiles to be in good agreement with experimental and amongst its one- and two-dimensional representations.

## Introduction

CICR is the repeated process in which influx of extracellular Ca^2+^ into the cytosol from L-type channels triggers a release of Ca^2+^ from the sarcoplasmic reticulum (SR) through ryanodine receptors (RyRs). Ca^2+^ releases from the SR into the cytosol appear as sparks in the cytosol and as blinks in the SR. The successive activation of RyRs increases the local Ca^2+^ concentration in the cytosol. This process is called Ca^2+^ transient and the sequential activation of Ca^2+^ results in spontaneous propagating waves of Ca^2+^ which occur in ventricular cardiac myocytes mainly due to Ca^2+^ overload. This occurs under pathological conditions affecting the heart’s normal function which may lead to heart failure and ventricular arrythmias (Lakatta and Guarnieri 1993).

Ca^2+^ sparks in the heart muscle was observed to behave stochastically (Cannell et al. 1995) and that Ca^2+^ releases occur at discrete sites with regular spacing of 1.8–2 µm longitudinally and more irregular spacing with a mean value of 0.76 µm transversely (Parker et al. 1996; Shacklock et al. 1995). Spontaneous Ca^2+^ sparks were accounted for in several mathematical models as either deterministic or stochastic. The first noteworthy model accounts for Ca^2+^ release at uniformly distributed sites and releases follow a time dependent process with an exponential rise and fall of the Ca^2+^ flux from the SR (Backx et al. 1989). The CICR process is described by a deterministic formulation whereby the release is dependent on a threshold Ca^2+^ concentration. The process is represented by a one-dimensional model accounting only for longitudinal diffusion.

Other similar models (Smith et al. 1998; Izu et al. 2001a; Subramanian et al. 2001) that also include a deterministic formulation of the Ca^2+^ releases, represent the system with a two-dimensional model whereby spatial and anisotropic properties of the Ca^2+^ diffusion can be successfully reproduced. The models mentioned above account for free fluorescent indicator dye and Ca^2+^-binding proteins such as endogenous buffers. Additionally, the system was based on Fick’s Law diffusion derived using a system of partial differential equations (PDEs) and ordinary differential equations (ODEs). Ca^2+^ sparks are stochastic in nature (Cannell et al. 1995) and this has since been included in models (Chen et al. 2013, 2014, 2018a, b; Arif et al. 2019; Izu et al. 2001b; Li et al. 2010).

The use of fluorescent indicator dye and buffer properties are an integral part of studying cardiac myocyte physiology in health and disease. Not only does the use of dye and buffers in experimental studies of cardiac myocytes enable the observation and measurement of Ca^2+^ wave properties, but also it can have a direct influence on the wave dynamics. Studies on eukaryotic cells (Wang and Thompson 1995), Xenopus oocytes (Jafri and Keizer 1995) and astrocytes (Wang et al. 1997) using indicator fura-2 shows measures that immobile/mobile buffers slow down the propagation of Ca^2+^ waves as well as reduces the wave amplitudes. In a more recent study (Bovo et al. 2015) on the effect of buffering in cardiac myocytes, fluo-4 and fluo-5 N were used to measure sparks and blinks, respectively. It has been shown that the presence of Ca^2+^ buffers (more specifically BAPTA) has supressed Ca^2+^ spark activity as well as caused earlier termination of these sparks. Nevertheless, the effect of different types and level of buffering and dye properties on the Ca^2+^ dynamics is not considered to be fully explored. This is especially important since these properties may influence calcium handling and contractility (Smith and Eisner 2019).

Majority of stochastic models mainly explore the Ca^2+^ movement in the cytosol. However, Ca^2+^ sparks in the cytosol are directly linked to Ca^2+^ blinks in the SR. To account for such coupling link, a system of coupled differential equations must be formulated. One such model has been formulated (Chen et al. 2018b; Li et al. 2017) with either a partial differential equation (PDE) or an ordinary differential equation (ODE) representing the Ca^2+^ movement in the SR. All models mentioned have greatly improved our understanding of the Ca^2+^ dynamics under pathological conditions. However, more exploration is needed to gain new insight into the coupled system. The idea of using a coupled system of PDEs or a system of partial and ordinary differential equations for modelling this coupling linkage is not novel. The choice of equations in the coupling link is vital towards a better estimate of the Ca^2+^ sparks and blinks. This needs further investigation as to gain insight into the drawbacks and trade-offs of using such models. In the current work, there are two goals set out to achieve. One of them is to extend our investigation from our previous work (Serife et al. 2017) and gain new insight into modelling the cytosolic and sarcoplasmic Ca^2+^ sparks and blinks using coupled PDEs. The second goal is to formulate a fully coupled system with stochastic Ca^2+^ release that not only reproduces the cytosolic Ca^2+^ sparks but also produces the sarcoplasmic Ca^2+^ blinks comparable to those experimentally observed. Finally, the third and last goal of this work is to observe the effects of cytosolic and sarcoplasmic buffers and dye on the speed and amplitude of Ca^2+^ wave propagation.

## Methods

### Model for stochastic calcium waves

The model presented in the current work follows from our previous work (Serife et al. 2017) on the stochastic extension of the deterministically coupled model (Dupont and Goldbeter 1994, 1997; Tracqui and Ohayon 2009). The governing equations for Ca^2+^ sparks and blinks are based on the Fick’s Law diffusion for simplicity and are given in Eqs. (1) and (2) as below.

1 $$\frac{\partial C}{\partial t}=\;{D}_{Cx}\frac{{\partial }^{2}C}{\partial {x}^{2}}+{D}_{Cy}\frac{{\partial }^{2}C}{\partial {y}^{2}}+{J}_{dye}+{J}_{buffer}-{J}_{pump}-{J}_{C{a}^{2+}leak}+{J}_{CRU}+{J}_{SRleak,}$$

2 $$\frac{\partial {C}_{S}}{\partial t}=\;{D}_{{C}_{S}x}\frac{{\partial }^{2}{C}_{S}}{\partial {x}^{2}}+{D}_{{C}_{S}y}\frac{{\partial }^{2}{C}_{S}}{\partial {y}^{2}}+{J}_{dye}+{J}_{buffer}+{J}_{pump}-{J}_{CRU}-{J}_{SRleak.}$$

The cytosolic and sarcoplasmic Ca^2+^ concentrations are represented by $C$ and ${C}_{S}$, respectively. ${D}_{Ci}$, ${D}_{{C}_{S}i}$, $i=x,y$ are cytosolic and sarcoplasmic diffusion coefficients in the *x* and *y* directions; ${J}_{C{a}^{2+}leak}$ and ${J}_{SR leak}$ represent the cytosolic Ca^2+^ leak from the cytosol to the exterior of the cell and sarcoplasmic Ca^2+^ leak from the SR to the cytosol; ${J}_{pump}$ represents the pumping rate of SR Ca^2+^-ATPase. The last three terms are given as ${J}_{C{a}^{2+}leak}=kC$, ${J}_{SR leak}={k}_{f}{C}_{S}$ and ${J}_{pump}={V}_{pump} {C}^{{n}_{pump} }/({K}_{pump}^{{n}_{pump}}+{C}^{{n}_{pump}}),$ where ${V}_{pump}$ is the maximum rate, ${K}_{pump}$ is the affinity constant for SR pumps and ${n}_{pump}$ is the Hill constant with $k$ and ${k}_{f}$ representing rates of passive Ca^2+^ efflux from the cytosol to the extracellular medium and from the SR into the cytosol.

The flux of Ca^2+^ release from a Ca^2+^ release unit (CRU) is determined by ${J}_{CRU}=\sigma \sum {\delta }_{D}(x-{x}_{\alpha }){\delta }_{D}(y-{y}_{\beta })S(x,y,t;{T}_{open})$ where $\sigma =\frac{{I}_{CRU}}{2{F}_{c}}$ is a molar flux from a Ca^2+^ release channel based on the current of the CRU, ${I}_{CRU}$, and Faraday’s constant, ${F}_{c}$. The Dirac delta, ${\delta }_{D}$, term in the summation represents a point source (i.e. the CRU) located at ${x}_{\alpha }$, ${y}_{\beta }$. ${J}_{dye}$ and ${J}_{buffer}$ are fluxes due to the Ca^2+^ fluorescent indicator dye and endogenous stationary buffers. These are given as ${J}_{buffer}=-\frac{d[CaB]}{dt}$ and ${J}_{dye}=-{k}_{f}^{+}C([F{]}_{T}-[CaF])+{k}_{f}^{-}[CaF]$ where ${\left[F\right]}_{T}$ is the total concentration of dye, and ${k}_{f}^{+}$ and ${k}_{f}^{-}$ are forward and reverse rate constants for dye. The Ca^2+^ bound dye, $[CaF]$, and Ca^2+^ bound buffer, $[CaB]$, are assumed to be mobile and immobile (Izu et al. 2001a) and, thus, are updated using a PDE and ODE, respectively. These are given in Eqs. (3) and (4) as below.

3 $$\frac{\partial [CaF]}{\partial t}={D}_{Dx}\frac{{\partial }^{2}[CaF]}{\partial {x}^{2}}+{D}_{Dy}\frac{{\partial }^{2}[CaF]}{\partial {x}^{2}}-{J}_{dye,}$$

4 $$\frac{d[CaB]}{dt}={k}_{B}^{+}C(\left[B{]}_{T}-\left[CaB\right]\right)-{k}_{B}^{-}\left[CaB\right],$$

where ${D}_{Dx}$ and ${D}_{Dy}$ are diffusion coefficients for fluorescent dye. Similarly, ${k}_{B}^{+}$ and ${k}_{B}^{-}$ are forward and reverse rate constants for buffer, and $[B{]}_{T}$ is the total buffer concentration. The firing of CRUs is controlled by the stochastic function$S$. The probability that a release unit fires is $P(C(x,y,t),{K}_{prob},{n}_{prob}).\Delta t$ where $P(C(x,y,t),{K}_{prob},{n}_{prob})={P}_{max}\frac{[C{a}^{2+}{]}^{{n}_{prob}}}{({K}_{prob}+[C{a}^{2+}{]}^{{n}_{prob}})}$. ${P}_{max}$ is the maximum probability of Ca^2+^ spark occurrence, ${K}_{prob}$ is the Ca^2+^ sensitivity parameter and ${n}_{prob}$ is the Hill coefficient. The stochastic opening of a release unit is stimulated at each time interval $\Delta t$ by generating a uniformly distributed random number ${u}_{r}$ between 0 and 1. As$C(x,y,t)\to \infty$, $\frac{P}{{P}_{max}} \to 1$ so the probability that ${u}_{r}$ is less than $\frac{P}{{P}_{max}}$ is less than 1. Therefore, in the case where ${u}_{r}<\frac{P}{{P}_{max}}$, the release unit fires by setting $S=1$ and the unit remains open for ${T}_{open}$ ms. Once the channel is closed it remains closed. In the case where${u}_{r}>\frac{P}{{P}_{max}}$, the release unit does not open and remains closed until${u}_{r}<\frac{P}{{P}_{max}}$.

### Improved sarcoplasmic buffer and dye properties

Ca^2+^ sparks and blinks are two different views of the same elementary release events. Ca^2+^ blinks are important events for the termination of sparks. An ideal model should be able to estimate the fluorescent values in the cytosol as well as that in the SR. In the previous section, the fluorescent dye and buffers in the SR are not taken into account. Modelling of such coupling has not been fully explored. Therefore, model presented in the previous section is modified and extended to accommodate for an improved estimation of the Ca^2+^ blinks with the normalised fluorescent profile in the SR. In addition to the equations used in the previous section, new equations are introduced for estimating the sarcoplasmic Ca^2+^ concentration, Ca^2+^ bound fluorescent indicator, $[CaF]$, and Ca^2+^ bound buffer, $[CaB]$, in the SR. The governing equation for the sarcoplasmic Ca^2+^ concentration in Eq. (2) is modified to Eq. (5) as below.

5 $$\frac{\partial {C}_{s}}{\partial t}=\;{D}_{{C}_{s}x}\frac{{\partial }^{2}{C}_{s}}{\partial {x}^{2}}+{D}_{{C}_{s}y}\frac{{\partial }^{2}{C}_{s}}{\partial {y}^{2}}+{J}_{SR dye}+{J}_{SR buffer}+{J}_{pump}-{J}_{SR leak}+{J}_{RyR},$$

where the terms ${J}_{SR dye}$, ${J}_{SR buffer}$, and ${J}_{RyR}$ are defined as (Li et al. 2017; Tan et al. 2007) ${J}_{SR dye}=-{K}_{F}^{+}{C}_{s}\left({\left[F\right]}_{{T}_{SR}}-{\left[CaF\right]}_{SR}\right)+{K}_{F}^{-}{\left[CaF\right]}_{SR}$, ${J}_{SR buffer}=-\frac{d{\left[CaB\right]}_{SR}}{dt}$ and ${J}_{RyR}={\sigma }_{RyR}\sum_{\alpha , \beta }{\delta }_{D}\left(x-{x}_{\alpha }\right){\delta }_{D}\left(y-{y}_{\beta }\right)S\left(x,y,t;{T}_{open}\right),$ where ${\left[CaF\right]}_{SR}$ is spatially and temporally diffusive, ${\left[CaB\right]}_{SR}$ is only diffusive in time (Li et al. 2017). These two are therefore updated with a PDE and an ODE (Li et al. 2017) as given in Eqs. (6) and (7) as below.

6 $$\frac{\partial {\left[CaF\right]}_{SR}}{\partial t}={D}_{F}\left(\frac{{\partial }^{2}{\left[CaF\right]}_{SR}}{\partial {x}^{2}}+\frac{{\partial }^{2}{\left[CaF\right]}_{SR}}{\partial {y}^{2}}\right)-{J}_{SR dye,}$$

7 $$\frac{d{\left[CaB\right]}_{SR}}{dt}={K}_{CSQ}^{+}{C}_{s}\left({\left[B\right]}_{{T}_{SR}}-{\left[CaB\right]}_{SR}\right)-{K}_{CSQ}^{-}{\left[CaB\right]}_{SR.}$$

The molar flux, ${\varvec{\sigma}}$, in the SR is governed by the current through the SR channels, ${{\varvec{I}}}_{{\varvec{R}}{\varvec{y}}{\varvec{R}}}$, grid sizes in the x and y directions, $\boldsymbol{\Delta }{\varvec{x}}$ and $\boldsymbol{\Delta }{\varvec{y}}$, and the discrepancy between the cytosolic and sarcoplasmic Ca^2+^ concentrations (Li et al. 2017), ${\left[{\varvec{C}}{{\varvec{a}}}^{2+}\right]}_{{\varvec{c}}{\varvec{y}}{\varvec{t}}}-{\left[{\varvec{C}}{{\varvec{a}}}^{2+}\right]}_{{\varvec{S}}{\varvec{R}}}$, as ${{\varvec{\sigma}}}_{{\varvec{R}}{\varvec{y}}{\varvec{R}}}=\frac{{{\varvec{I}}}_{{\varvec{R}}{\varvec{y}}{\varvec{R}}}}{2{{\varvec{F}}}_{{\varvec{c}}}\boldsymbol{\Delta }{\varvec{x}}\boldsymbol{\Delta }{\varvec{y}}}\left({\left[{\varvec{C}}{{\varvec{a}}}^{2+}\right]}_{{\varvec{c}}{\varvec{y}}{\varvec{t}}}-{\left[{\varvec{C}}{{\varvec{a}}}^{2+}\right]}_{{\varvec{S}}{\varvec{R}}}\right)$.

### Numerical methods

A computational domain with dimensions 20 × 100 µm is used meshed with a grid size of 0.8/3 × 0.4 µm and time step size of 0.003125 ms. Channels are spaced ${l}_{x}=2\mathrm{\mu m}$ and ${l}_{y}=0.8\mathrm{\mu m}$ apart longitudinally and vertically, respectively (Table 1). As boundary conditions zero-flux is assumed at the cell boundaries by imposing $\partial C/\partial x=\partial C/\partial y=0$ and $\partial {C}_{S}/\partial x=\partial {C}_{s}/\partial y=0$ (Backx et al. 1989; Smith et al. 1998; Izu et al. 2001a, 2001b; Chen et al. 2013, 2014; Serife et al. 2017). Similar assumption is imposed on the Ca^2+^ bound dye, i.e. $\partial \left[CaF\right]/\partial x=\partial [CaF]/\partial y=0$ (Backx et al. 1989; Smith et al. 1998; Izu et al. 2001a, 2001b; Chen et al. 2013, 2014; Arif et al. 2019; Serife et al. 2017). The governing Eqs. (1), (2) and (3) in one dimension are discretised using the Crank–Nicolson finite difference method. Equation (4) is discretised and solved using the forward Euler method. In the two-dimensional case, Eqs. (1), (2) and (3) are discretised using the alternating direction implicit (ADI) method. Discretising Eq. (2) is given in two steps as in Eqs. (8) and (9) below.

8 $$-\frac{{D}_{Dx}\Delta t}{2\Delta {x}^{2}}[{C}_{S}{]}_{i-1j}^{n+\frac{1}{2}}+\left(1+\frac{{D}_{Dx}\Delta t}{\Delta {x}^{2}}+\frac{{k}_{f}}{2}\Delta t\right)[{C}_{S}{]}_{ij}^{n+\frac{1}{2}}-\frac{{D}_{Dx}\Delta t}{2\Delta {x}^{2}}=\left(\frac{1}{2}{D}_{Dy}{\delta }_{y}^{2}+1\right)[{C}_{S}{]}_{ij}^{n}+\frac{1}{2}\left(T\right),$$

9 $$-\frac{{D}_{Dy}\Delta t}{2\Delta {y}^{2}}[{C}_{S}{]}_{ij-1}^{n+1}+\left(1+\frac{{D}_{Dy}\Delta t}{\Delta {y}^{2}}+\frac{{k}_{f}}{2}\Delta t\right)[{C}_{S}{]}_{ij}^{n+1}-\frac{{D}_{Dy}\Delta t}{2\Delta {y}^{2}}[{C}_{S}{]}_{ij+1}^{n+1}=\left(\frac{1}{2}{D}_{Dx}{\delta }_{x}^{2}+1\right)[{C}_{S}{]}_{ij}^{n+\frac{1}{2}}+\frac{1}{2}\left(T\right),$$

where the additional terms are $T=\left({{J}_{dye}}^{n}+{{J}_{buffer}}^{n}+{{J}_{pump}}^{n}-{{J}_{CRU}}^{n}\right)$ and the ${\delta }_{x}^{2}$, ${\delta }_{y}^{2}$ correspond to the standard second order central differences in the x and y directions, respectively. These are given as ${\delta }_{x}^{2}[{C}_{S}{]}^{n}=\frac{[{C}_{S}{]}_{i-1j}^{n}-2[{C}_{S}{]}_{ij}^{n}+[{C}_{S}{]}_{i+1j}^{n}}{\Delta {x}^{2}}$ and ${\delta }_{y}^{2}[{C}_{S}{]}^{n}=\frac{[{C}_{S}{]}_{ij-1}^{n}-2[{C}_{S}{]}_{ij}^{n}+[{C}_{S}{]}_{ij+1}^{n}}{\Delta {y}^{2}}$.

**Table 1:** Standard parameter values

Symbol	Values	Units
${D}_{Cx}$,${D}_{Cy}$	200	µm^2^s^−1^
${D}_{Dx}$,${D}_{Dy}$	20	µm^2^s^−1^
${D}_{{C}_{s}x}$,${D}_{{C}_{s}y}$	12	µm^2^s^−1^
${l}_{x}$,${l}_{y}$	2,0.8	µm
$[C{a}^{2+}{]}_{cyt(\infty )}$	0.1	µM
$[C{a}^{2+}{]}_{SR(\infty )}$	640	µM
${F}_{c}$	96,500	Cmol^−1^
${V}_{pump}$	0.208	µMs^−1^
${K}_{pump}$	0.184	µM
${K}_{prob}$	15	µM
${n}_{pump}$,${n}_{prob}$	3.9,1.6	µM
$k$,${k}_{f}$	10,1	s^−1^
${P}_{max}$	0.3	s^−1^
${I}_{CRU}$	20	pA
${k}_{B}^{+}$,${k}_{f}^{+}$	100,80	(µMs)^−1^
${k}_{B}^{-}$,${k}_{f}^{-}$	100,90	s^−1^
${\left[B\right]}_{T}$,${\left[F\right]}_{T}$	123,50	µM

Equations (1) and (3) are discretised as before in Serife et al. (2017) and will not be given here. The discretised equations are then represented by a matrix and vector form where a tridiagonal matrix solver is used to numerically solve this system of equations. For the model with an improved sarcoplasmic calcium dye and buffer representation, Eqs. (1) and (3)–(7) are discretised using the same methods mentioned above. For the one-dimensional case, a combination of Crank–Nicolson finite difference method for the PDEs and Euler method for the ODEs. As for the two-dimensional case, a combination of ADI for the PDEs and Euler method for the ODEs. The non-buffered and non-dyed version of this improved model is also treated in a similar fashion by using the mentioned models depending on the dimensionality. The simulation results are given in the next section.

## Results

### Comparison of the one- and two-dimensional models

**Fig. 1:**
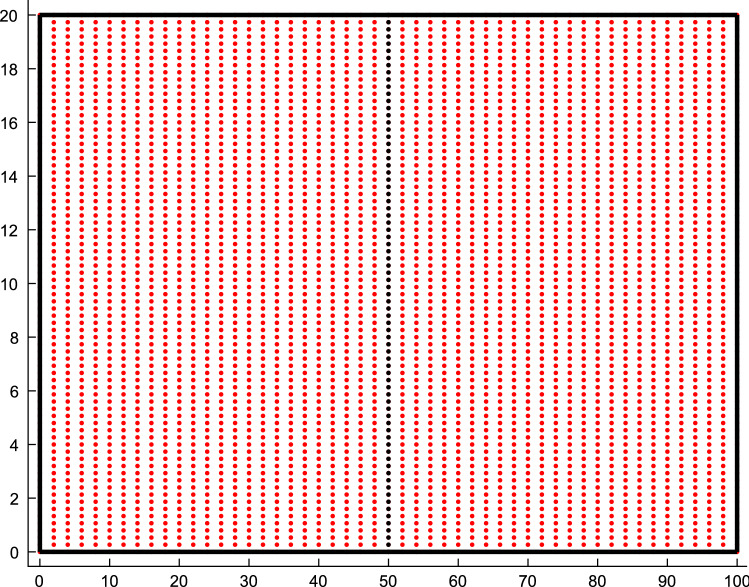
The uniform distribution of the CRU channels on the Cartesian domain for the two-dimensional case. Two columns of point sources vertically (black dotted points) along the middle of the domain (i.e. at 50 µm and 50.4 µm) are force activated to initiate a wave

**Fig. 2:**
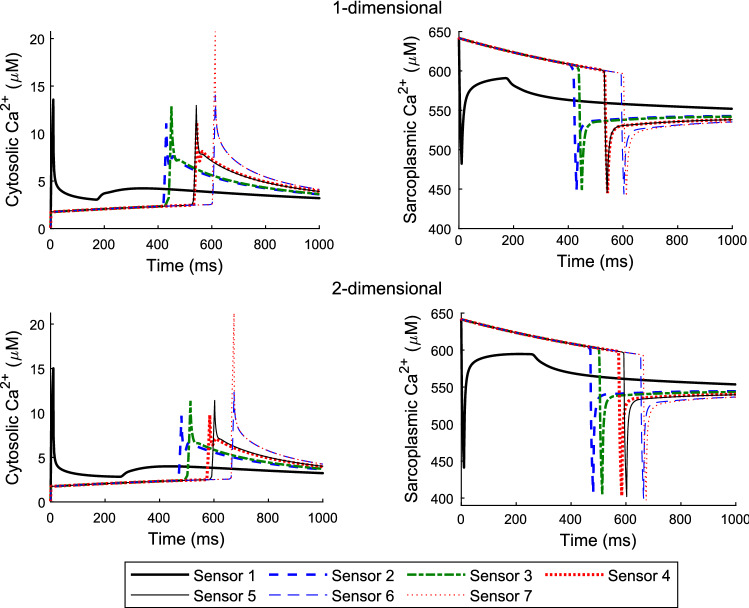
One-dimensional (top panels) and two-dimensional (bottom panels) model results for the cytosolic (left panels) and sarcoplasmic (right panels) Ca^2+^ wave formation from initiated sparks and blinks with cytosolic Ca^2+^ buffer and dye included. Sensors correspond to probes placed at locations to record computed values of cytosolic and sarcoplasmic calcium. Sensors 1–7 are located at 0, 25, 25, 38, 38, 49 and 50 µm longitudinally from the source of the initially released calcium and 9.6 µm in the transverse direction for the two-dimensional case (See Fig. 1 for how the domain is set up). Ca^2+^ sparks and blinks are initiated at the centre of the cell domain as shown above by the first peaks and blinks. Peak (cytosolic) concentration values at sensors 1–7 in µM for the one- and two-dimensional models are (13.58, 11.07, 12.86, 11.19, 12.96, 14.08, 20.74) and (15.03, 9.70, 11.36, 9.77, 11.41, 12.37, 21.23), respectively. Trough (sarcoplasmic) values in µM at sensors 1–7 for the one- and two-dimensional models are (482.2, 448.8, 448.8, 445, 445.9, 443.4, 441) and (441.1, 406.6, 405, 403, 402, 399.3, 397), respectively

The two-dimensional system is set to be isotropic (${D}_{Cx}={D}_{Cy}$, ${D}_{{D}_{x}}={D}_{{D}_{y}}$ and ${D}_{{C}_{s}x}={D}_{{C}_{s}y}$) for the purpose of comparing with the one-dimensional system. The calcium release is set to be based on two columns of point sources in the middle of the domain (Fig. 1). The source strengths, $\sigma$, for both the one- and two-dimensional models require separate numerical conversions since a line source is not equivalent to a linear array of discrete CRUs. Therefore, the molar flux of the line source requires adjustment to approximate the molar flux of a point source. The source strengths used are ${\sigma }_{1}=0.19{\sigma }_{3}$ and ${\sigma }_{2}=0.51{\sigma }_{3}$ for the one- and two-dimensional systems, respectively (See appendix and (Serife et al. 2017) for derivation). ${\sigma }_{i}, i=\mathrm{1,2},3$ refer to the source strength in one-, two- and three-dimensional settings. Solving Eqs. (1)–(4) in both one dimension (i.e. derivatives in the y-direction are set to zero) and two dimensions with the parameters set to values in Table 1 (unless mentioned) (Izu et al. 2001a, 2001b; Chen et al. 2013, 2014) give the results in Fig. 2.

Figure 2 shows the cytosolic and sarcoplasmic Ca^2+^ concentrations for the one-dimensional (top panels) and two-dimensional (bottom panels) models, respectively. From initiation to activation of neighbouring channels there is some delay for Ca^2+^ near the neighbouring channels to reach activation threshold. Once the neighbouring channels are activated, there is an increase in Ca^2+^ around the region which explains the decrease then slight increase in concentration from spark initiation. The results for the sarcoplasmic Ca^2+^ shows a drop to between 441 and 482.2 µM for the one-dimensional model and between 397 and 441.1 µM for the two-dimensional case. This is a large reduction in the differences between the two models compared to our previous work (Serife et al. 2017) where an ODE is used instead of a PDE to represent the sarcoplasmic Ca^2+^ concentration. Comparing results for the one- and two-dimensional models in our previous work (Serife et al. 2017) showed large discrepancies in the sarcoplasmic Ca^2+^ concentrations between the one- and two-dimensional models of about 350 µM. With this modified model this discrepancy has reduced to approximately 40 µM.

**Fig. 3:**
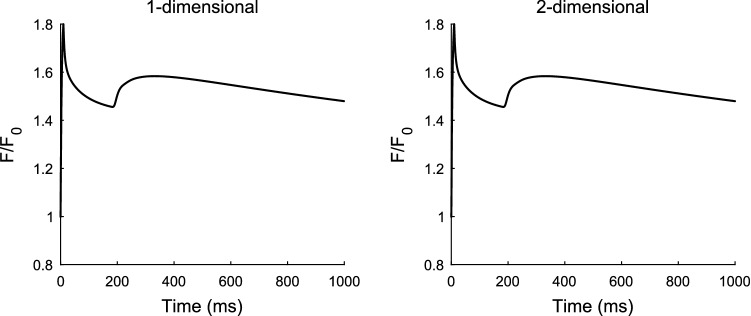
Normalised cytosolic fluorescent dye profile for the one-dimensional model (left) and two-dimensional model (right). There is a similar pattern of decrease then slight increase in profile just after initiating a spark. This is due to increase in concentration around the region where neighbouring channels are activated leading to additional Ca^2+^ release near the first initiation point

On the other hand, the time course of the cytosolic Ca^2+^ waves are consistent between the two models as apparent in Fig. 2 (left panels) and Fig. 3 where the normalised cytosolic fluorescent dye profiles, F/F0, are given for the two models showing a peak amplitude of 1.8 in both cases.

### Improved sarcoplasmic buffer and dye properties: comparison of one- and two-dimensional models

Similar to the previous section the two-dimensional model is set to be isotropic (${{\varvec{D}}}_{{\varvec{C}}{\varvec{x}}}={{\varvec{D}}}_{{\varvec{C}}{\varvec{y}}}$*,* ${{\varvec{D}}}_{{{\varvec{D}}}_{{\varvec{x}}}}={{\varvec{D}}}_{{{\varvec{D}}}_{{\varvec{y}}}}$ and ${{\varvec{D}}}_{{{\varvec{C}}}_{{\varvec{s}}}{\varvec{x}}}={{\varvec{D}}}_{{{\varvec{C}}}_{{\varvec{s}}}{\varvec{y}}}$). The one- and two-dimensional models are discretised in a similar fashion to the ones before using CN for the one-dimensional PDEs and ADI for the two-dimensional PDEs with forward Euler used for the ODEs. The parameters associated with the rate of passive Ca^2+^ efflux, k and kf, are set to 8 s^−1^ and 0.25 s^−1^, respectively. The sarcoplasmic Ca^2+^ at rest is set to 954 µM. The parameters associated with the sarcoplasmic buffers and dye used are given in Table 2 (Li et al. 2017). The rest of the parameters not mentioned here are adopted from the previous section (Table 1).

**Table 2:** Standard parameter values for sarcoplasmic buffer and dye

Symbol	Values	Units
${D}_{F}$	1.6	µm^2^s^−1^
${\left[Ca2+\right]}_{SR(\infty )}$	954	µM
${\left[B\right]}_{{T}_{SR}}$, ${\left[F\right]}_{{T}_{SR}}$	0.014, 20	µM
${K}_{CSQ}^{+}$, ${K}_{CSQ}^{-}$	100, 60,000	µMs^−1^, s^−1^
${K}_{F}^{+}$, ${K}_{F}^{-}$	48.8, 19,520	µMs^−1^, s^−1^
${I}_{RyR}$	0.07	pA

**Fig. 4:**
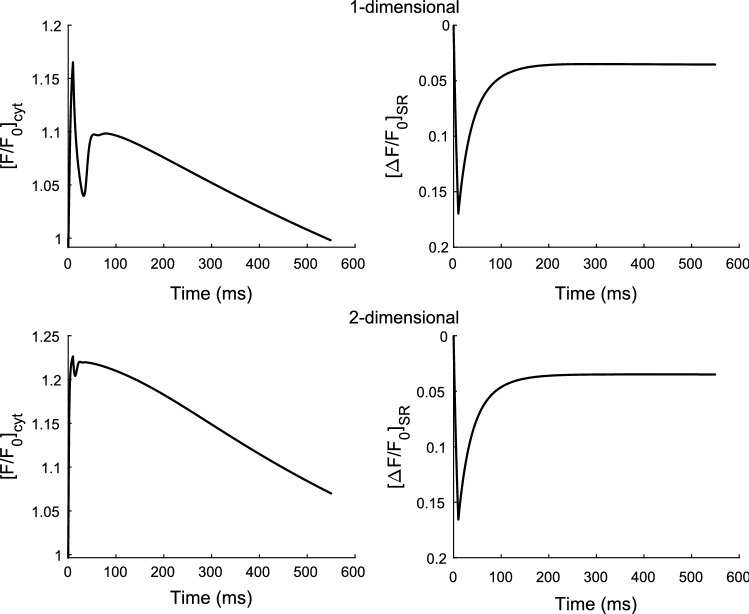
Normalised cytosolic fluorescent dye profiles (left panels) and normalised sarcoplasmic fluorescent dye profiles (right panels) for the one-dimensional model (top panels) and two-dimensional model (bottom panels)

Figure 4 shows the simulation results run for 550 ms for reproducing the time course of the normalised cytosolic (left panels) and sarcoplasmic (right panels) fluorescent indicator dye profiles for the one-dimensional (top panels) and two-dimensional (bottom panels) models. Observing the normalised fluorescent values in the SR, i.e. ${\left[\boldsymbol{\Delta }{\varvec{F}}/{{\varvec{F}}}_{0}\right]}_{{\varvec{S}}{\varvec{R}}}$ profile, the drop is computed to be 0.16 which represents the blink amplitude. Although the blink amplitudes in both the one- and two-dimensional cases match closely, there is a discrepancy in the normalised cytosolic fluorescent dye values of about 0.6 with a value of 1.17 in the one-dimensional and 1.23 in the two-dimensional cases.

The estimated blink amplitude of 0.085 is comparable to a previously published numerical study (Li et al. 2017) and experimentally observed blink amplitude of 0.08 ± 0.02 (Brochet et al. 2005). However, the experimentally observed range has been reported to be an underestimate of the true Ca^2+^ depletion and a corrected blink amplitude (i.e. ${\left[\Delta F/{F}_{0}\right]}_{SR}$) is measured as 0.13 (Brochet et al. 2005) in which the computed blink values in this work is a close estimate.

### Non-buffered and non-dyed properties

**Fig. 5:**
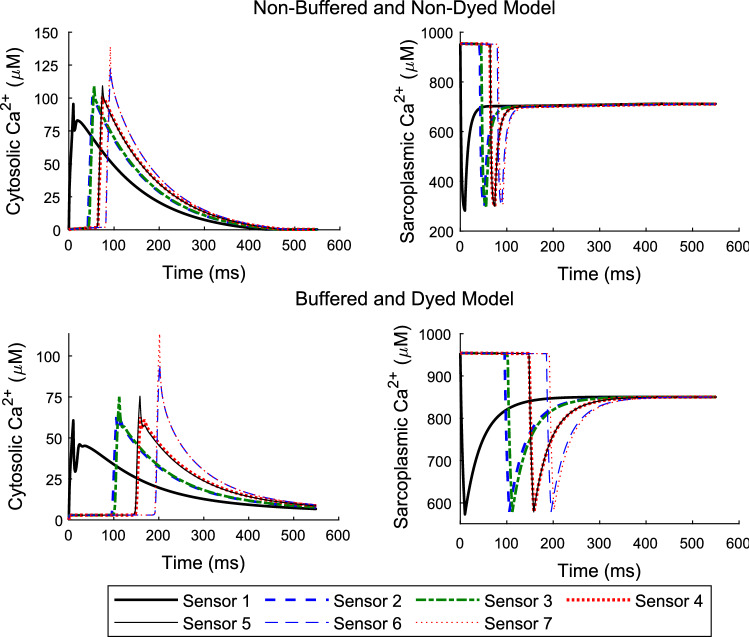
Cytosolic and sarcoplasmic Ca^2+^ concentration results in the absence of buffer and dye (top panels) and that in the presence of buffer and dye properties (bottom panels). As mentioned before sensors correspond to probes placed at locations to enable the time course of computed concentrations to be recorded. Sensors 1–7 are located at 0, 25, 25, 38, 38, 49 and 50 µm longitudinally from the source of the initially released calcium and 9.6 µm in the transverse direction for the two-dimensional case (See Fig. 1 for how the domain is set up). Peak (cytosolic) concentration values in µM at sensors 1–7 are (95.54, 100.9, 109.2, 101.3, 109.3, 122.4, 139) for the non-buffered and non-dyed model, and for the buffered and dyed model are (60.76, 62.54, 75.12, 62.7, 75.46, 95.43, 113.6), respectively. Trough (sarcoplasmic) values in µM at sensors 1–7 are (282.1, 299.3, 299.5, 299.1, 299.3, 301.2, 309.5) for the buffered and dyed model, and for the non-buffered and non-dyed model are (572.8, 578.9, 578.7, 578.6, 578.7, 578.6, 583.9), respectively

This section is aimed at giving the simulation results on the cytosolic and sarcoplasmic Ca^2+^ wave propagation in the absence of buffer and dye properties in comparison with when buffer and dye are present. The model presented in Sect. 3.2 is used to distinguish the effects of buffer and dye on the Ca^2+^ movement in both the cytosol and the SR compartments by setting ${J}_{buffer}={J}_{dye}=0$ and ${J}_{SR buffer}={J}_{SR dye}=0$ whilst keeping all other parameters constant. Figure 5 shows the results for solving the model in 3.2 with (bottom panels) and without (top panels) cytosolic and sarcoplasmic buffers and dye.

The parameters in this model reflect fluo-3 used in the cytosol and fluo-5 N used in the SR compartments. The results show a noticeable change in both amplitude and speed of Ca^2+^ movement in the cytosol and SR. The termination of Ca^2+^ waves take twice as long with the presence of buffers and dye than that without these properties. The cytosolic Ca^2+^ peaks are supressed in the presence of buffers and dye roughly by 35%. This inevitably changes the amount of depletion in the SR. The amount depleted has dropped by approximately 57% when buffers and dye are present.

## Discussion and conclusion

Comparing results of the one- and two-dimensional models in our previous work (Serife et al. 2017) showed large discrepancies in the sarcoplasmic Ca^2+^ concentrations between the models of about 350 µM. The model formulation for the sarcoplasmic Ca^2+^ was limited to an ODE. In this work, the ODE is replaced by a PDE and the discrepancy in the sarcoplasmic Ca^2+^ concentrations between the two cases has decreased to 40 µM. The large discrepancy is due to the spacing between channels in the y-direction introducing a large variation in the SR Ca^2+^ content. Given this observation when the Ca^2+^ movement in the SR is modelled using an ODE the dispersive nature is not accurately simulated. Therefore, the use of a PDE becomes more important.

Another important observation made which will be very useful when modelling such dynamic behaviour in cardiac myocytes using DEs is the results from the modified and extended model given in the previous section that accounts for the Ca^2+^ bound buffer and dye movements in the SR. The blink amplitude obtained here shows good agreement with results obtained in the experimental (Brochet et al. 2005) and computational (Li et al. 2017) studies. On the other hand, the results obtained for the normalised fluorescent dye in the cytosol shows slight discrepancy from the one-dimensional to the two-dimensional model. This shows that a source strength adjustment that was made for the cytosolic Ca^2+^ release molar flux may also be needed in the case for the sarcoplasmic Ca^2+^ molar flux.

The points discussed above are important to consider when modelling the Ca^2+^ movement in cardiac myocytes using DEs since most often ODEs are used as simplification and as a consequence, one must keep in mind the resulting trade-offs. If an ODE is used in replacement of a PDE for estimating the cytosolic Ca^2+^ movement this might be acceptable given that one is aware of the large discrepancies in the Ca^2+^ content and useful insight is drawn from the cytosolic content. This point becomes more important as three-dimensional models become more frequent since the discrepancies can become larger due to the fact that spacing between channels in the z-direction will introduce additional variations into the cytosolic and sarcoplasmic Ca^2+^ contents.

Finally, the effect of buffers and dye based on the improved model given in this paper was also computationally investigated. The results showed that inclusion of buffers and dye, slows down the speed and suppresses the amplitude of Ca^2+^ wave propagation. This behaviour is observed in experimental studies using fura-2, fluo-3 (Wang and Thompson 1995; Wang et al. 1997) or a combination of fluo-4 and fluo-5 N (Bovo et al. 2015). However, the combined use of fluo-3 and fluo-5 N has not been explored in such a manner so it may not serve as a direct comparison. The study presented here can be further extended to explore the influence of fluo-5 N and fluo-3 indicators separately. This area remains not sufficiently investigated and future studies on distinguishing the effect of different types of dye and buffer properties will be beneficial to providing insight into the effects of these properties on Ca^2+^ movement and contractility.

Most mathematical models using stochastic partial differential equations, focus on studying the occurrence of individual Ca^2+^ sparks. The model presented here incorporates a stochastic activation of the CRUs which in turn effects the activation of the RyRs and is one of the few that couple stochastic cytosolic and sarcoplasmic Ca^2+^ producing fluorescent dye profiles comparable in both pattern and amplitude to that in experiments. In a previous model (Li et al. 2017), the stochastic coupling of the cytosolic and sarcoplasmic compartments was simultaneously solved, however, only results for the blinks were presented and the effect of blinks on the occurrence of sparks were not shown. The blink amplitude estimated previously (Li et al. 2017) as well as the model presented here produces a blink amplitude very close to the corrected blink amplitude in $\mathrm{\Delta F}/{\mathrm{F}}_{0}$ observed experimentally. Given the suitable parameters representing the Ca^2+^ bound buffer and dye reactions this model can easily be adapted for cell models for further evaluation. Previously, it was shown that using fractional differential equations (FDEs) as opposed to PDEs for modelling Ca^2+^ sparks and waves reproduces more accurate full-width at half-maximum (FWHM) property of the Ca^2+^ spark (Chen et al. 2013, 2014, 2018a, b; Tan et al. 2007; Li et al. 2011). A similar extension can be applied to the current work. The influx of Ca^2+^ from the extracellular medium to the cytosol occurs as regular pulses through L-type channels. The effect of this in the current work is addressed by including two columns of points sources in the middle of the domain which accounts for only one pulse, but this can be extended to regular intervals of pulses. Due to the focus of the work, this has not been accounted for.

## Appendix

An adjustment to the source strength was previously done (Serife et al. 2017) (adjusted from Izu et al. 2001b) to approximate the molar flux of a point source in two dimensions. This approach is extended for the one- and two-dimensional cases in this current work. The numerical conversion for the source strength involves the Green’s function obtained from the analytical solution of the diffusion problem for each of the dimensional cases and convolving this with the Heaviside function. The deduced relations for each of the cases are

$$C\left(x,t,1\right)=\frac{{\sigma }_{1}}{2D\sqrt{\pi }}\left(2D\sqrt{t}\mathrm{exp}{\left(-\frac{\Delta {x}^{2}}{{D}^{2}t}\right)}^{0.25}+\sqrt{\pi }\mathrm{erf}\left(\frac{\Delta x}{2D\sqrt{t}}\right)\right)$$

$$C\left(x,y,t,2\right)=\frac{{\sigma }_{2}}{4\pi D}{E}_{i}\left(\frac{{D}_{Cx}\Delta {y}^{2}+{D}_{Cy}\Delta {x}^{2}}{4{D}_{Cx}{D}_{Cy}t}\right)$$

$$C\left(x,y,z,t,3\right)=\frac{{\sigma }_{3}}{4\pi w}\mathrm{erf}\left(\frac{w}{2\sqrt{t}}\right),$$

where $w=\sqrt{\left({D}_{Cx}\Delta {y}^{2}+{D}_{Cy}\Delta {x}^{2}\right){D}_{Cz}+\Delta {z}^{2}{D}_{Cx}{D}_{Cy}}$ and $D$ is the geometric mean of ${D}_{Cx}$, ${D}_{Cy}$ and ${D}_{Cz}$. Equating the deduced expressions for the one- and two-dimensional cases and setting ${D}_{Cx}={D}_{Cy}={D}_{Cz}=0.2\mu {\mathrm{m}}^{2}{\mathrm{ms}}^{-1}$ with mesh sizes $\Delta x=0.4$, $\Delta y=0.8/3$ and $\Delta z=0.2$ and channel open duration ${T}_{open}=10\mathrm{ms}$ gives the relation ${\sigma }_{1}=0.19{\sigma }_{3}/\mu {\mathrm{m}}^{2}$ and ${\sigma }_{2}=0.51{\sigma }_{3}/\mu \mathrm{m}$ where ${\sigma }_{3}=\frac{{I}_{CRU}}{2F}$ which is incorporated into the simulation of the one- and two-dimensional models.
